# Accelerating brain T2-weighted imaging using artificial intelligence–assisted compressed sensing combined with deep learning-based reconstruction: a feasibility study at 5.0T MRI

**DOI:** 10.1186/s12880-025-01763-5

**Published:** 2025-07-01

**Authors:** Yun Wen, Huan Ma, Shaoxin Xiang, Zhichao Feng, Chuanjiang Guan, Xiang Li

**Affiliations:** 1 Department of Radiology, Chongqing University Three Gorges Hospital, Chongqing, 404000 China; 2 MR Collaboration, United Imaging Research Institute of Intelligent Imaging, Beijing, 100089 China; 3 United Imaging Healthcare Group, Shanghai, 201807 China

**Keywords:** Artificial intelligence–assisted compressed sensing, Deep learning, Magnetic resonance imaging, Compressed sensing, Brain

## Abstract

**Background:**

T2-weighted imaging (T2WI), renowned for its sensitivity to edema and lesions, faces clinical limitations due to prolonged scanning time, increasing patient discomfort, and motion artifacts. The individual applications of artificial intelligence-assisted compressed sensing (ACS) and deep learning-based reconstruction (DLR) technologies have demonstrated effectiveness in accelerated scanning. However, the synergistic potential of ACS combined with DLR at 5.0T remains unexplored. This study systematically evaluates the diagnostic efficacy of the integrated ACS-DLR technique for T2WI at 5.0T, comparing it to conventional parallel imaging (PI) protocols.

**Methods:**

The prospective analysis was performed on 98 participants who underwent brain T2WI scans using ACS, DLR, and PI techniques. Two observers evaluated the overall image quality, truncation artifacts, motion artifacts, cerebrospinal fluid flow artifacts, vascular pulsation artifacts, and the significance of lesions. Subjective rating differences among the three sequences were compared. Objective assessment involved the signal-to-noise ratio (SNR) and contrast-to-noise ratio (CNR) in gray matter, white matter, and cerebrospinal fluid for each sequence. The SNR, CNR, and acquisition time of each sequence were compared.

**Results:**

The acquisition time for ACS and DLR was reduced by 78%. The overall image quality of DLR is higher than that of ACS (*P* < 0.001) and equivalent to PI (*P* > 0.05). The SNR of the DLR sequence is the highest, and the CNR of DLR is higher than that of the ACS sequence (*P* < 0.001) and equivalent to PI (*P* > 0.05).

**Conclusions:**

The integration of ACS and DLR enables the ultrafast acquisition of brain T2WI while maintaining superior SNR and comparable CNR compared to PI sequences.

**Clinical trial number:**

Not applicable.

## Background

Magnetic resonance imaging (MRI) has emerged as an indispensable tool in neuroscience research, providing unprecedented insights into the complex relationships between brain structure, function, and cognitive processes, as well as their clinical implications in various neurological disorders [1, 2]. Among various imaging sequences, T2-weighted imaging (T2WI) has proven particularly valuable for edema and lesion detection, owing to its superior signal contrast and exceptional sensitivity to fluid accumulation [3]. However, the clinical application of T2WI is often limited by its inherent technical constraints. Specifically, the sequence requires extended echo time and repetition time, resulting in prolonged scan durations. This temporal limitation poses significant challenges in clinical practice, as patients may experience discomfort, claustrophobia, or involuntary movement during the examination, potentially compromising image quality through motion artifacts. To address these limitations while maintaining diagnostic efficacy, MRI protocols must carefully balance several critical parameters. The signal-to-noise ratio (SNR) and contrast-to-noise ratio (CNR) remain fundamental quality metrics in MRI system performance evaluation. Consequently, optimizing the trade-off between acquisition time and spatial resolution has become a crucial consideration in developing clinically viable imaging protocols.

In pursuit of reduced scanning durations, various accelerated imaging techniques, including parallel imaging (PI) and compressed sensing (CS), have been integrated into brain MRI schemes [4–6]. PI has become a mainstream clinical protocol that significantly reduces examination time and has had a substantial impact on neuroimaging. However, the benefits of PI in reducing examination time are constrained by the acceleration factor and the number of coil elements [7]. Another acceleration strategy, known as CS, has been introduced for incompletely filling k-space by capitalizing the data sparsity [8]. Unfortunately, the SNR of CS by simply reducing the k-space sampling may be lower than that of PI, limiting the acceleration effects in routine clinical practice [5].

Recent advancements in artificial intelligence (AI) have revolutionized MRI acquisition and reconstruction techniques, demonstrating transformative potential in clinical imaging [5]. In the field of image acquisition, a breakthrough methodology termed AI-assisted compressed sensing (ACS) has been developed through the synergistic integration of parallel imaging (PI), compressed sensing (CS), half Fourier (HF) acquisition, and convolutional neural networks (CNNs). This approach achieves unprecedented reductions in MRI acquisition time while preserving diagnostic fidelity, with a previous study focusing on fluid-attenuated recovery sequences in the brain confirming its effectiveness in shortening scan durations by up to 71% at a 3.0T MRI [9]. Concurrently, deep learning-based reconstruction (DLR) techniques have emerged as a powerful tool for intelligently recovering lost information by automatically leveraging available data [10]. By employing dual-domain processing that combines k-space filtering and image filtering techniques, DLR effectively discriminates between signals and noise, thereby optimizing both SNR and CNR. Altmann S et al. conducted a comprehensive evaluation of DLR at 1.5T MRI for acute stroke diagnosis, demonstrating that while the AI-accelerated brain MRI protocol achieved fourfold faster acquisition speeds, it maintained diagnostic equivalence with conventional MRI in detecting acute ischemic lesions [11]. While 1.5T/3.0T systems dominate clinical practice, 5.0T represents an emerging field strength that balances SNR gains and specific absorption rate (SAR) limitations compared to ultra-high-field systems (≥ 7.0T). Previous evidence supports the individual clinical value of ACS and DLR in the brain, but research on the compounded benefits of implementing these AI-driven approaches at a 5.0T field strength remains limited.

Therefore, the aim of this study was to systematically evaluate the diagnostic efficacy of the novel integrated ACS-DLR technique for T2WI acquisition at 5.0T through comparative analysis with conventional PI-based T2WI in patients with suspected cerebral conditions.

## Methods

### Patients

This prospective, single-center study was approved by the local ethics committee (KS-2024157). The study included patients undergoing brain MRI at our hospital from September 2024 to October 2024 who underwent both ACS-accelerated and conventional PI-accelerated T2WI sequences. The inclusion criterion was age ≥ 18 years. The exclusion criteria were technical non-synchronization between ACS and PI sequences, or patient refusal to participate.

### Image acquisition and image reconstruction

All examinations were conducted using a 5.0T MRI system (Jupiter, United Imaging Healthcare, Shanghai, China) equipped with a dedicated 48-channel head coil. Each participant underwent a standardized brain MRI protocol comprising both PI-accelerated T2WI (T2WI-PI) and ACS-accelerated T2WI (T2WI-ACS) sequences. Following T2WI-ACS acquisition, the DLR technique (uAIFI-DeepRecon, United Imaging Healthcare) was applied to generate T2WI-ACS-DLR sequences through offline post-processing of the raw ACS data on the magnetic resonance host. Detailed scanning parameters for T2WI-PI, T2WI-ACS, and T2WI-ACS-DLR are shown in Table 1. The acquisition time of the three protocols was recorded.

**Table 1:** Acquisition parameters of the conventional T2WI protocols and their ultrafast counterparts

Parameters	T2WI-PI	T2WI-ACS	T2WI-ACS-DLR
TR/TE (ms)	4805/114	4864/114	4864/114
Slice thickness(mm)	5	5	5
Slice gap(mm)	1.5	1.5	1.5
NEX	1.5	1	1
Field of view(mm)	230 × 200	230 × 200	230 × 200
Voxel(mm)	0.47 × 0.45 × 5	0.47 × 0.45 × 5	0.47 × 0.45 × 5
Acquisition time(sec)	159	35	35

Note. PI, parallel imaging; ACS, artificial intelligence-assisted compressed sensing; DLR, deep learning-based reconstruction; TR, repetition time; TE, echo time; NEX, number of excitations

The ACS technique employs a CNN to accelerate image acquisition processes [12]. To address the inherent uncertainties associated with CNN-based methods, ACS integrates the output of trained AI modules as additional constraints within the CS framework [13, 14]. This integration is achieved through the introduction of a regularization term that governs the discrepancy between reconstructed images and AI-predicted images. The ACS neural network was trained using a comprehensive dataset comprising two million fully sampled images acquired from phantoms (2%) and volunteers (98%) [12]. The iterative architecture design originated from k-space optimization, incorporating multi-scale sparsification strategies. This mathematical model synergistically combines essential components of compressed sensing, partial Fourier sampling, and parallel imaging techniques.

The DLR technology is based on a CNN model incorporating multiple enhancement modules and connection layers, utilizing an optimized lightweight neural network. In the intelligent processing of images, DLR adjusts parameters based on the original k-space image data while ensuring that the integrity of the original k-space is maintained, without impacting data acquisition and reconstruction. The DLR training dataset consists of millions of samples covering various anatomical structures and rich contrast scenes, intelligently mapping low-resolution, low-SNR original image data into high-resolution, high-SNR images with richer detail [15, 16]. To ensure the authenticity and reliability of the image details after deep learning, DLR incorporates a data fidelity module within the reconstruction chain. During network training, the loss function (evaluating the error rate of the training results) includes information from both the image domain and the k-space domain, constraining the intelligent module and strictly ensuring the authenticity of the network output results.

### Subjective image evaluation

All the images were anonymized. Two observers (with 7 and 12 years of experience in brain MRI interpretation, respectively), blinded to the three types of T2WI acceleration techniques, independently evaluated the images. A 5-point Likert scale was used to subjectively score the overall image quality, truncation artifact, motion artifact, cerebrospinal fluid flow artifact, vascular pulsation artifact, and the significance of lesions based on the signal intensity of the brain tissue and the image artifacts. A score of 5 was deemed the best image quality, while a score of 1 represented the poorest image quality (Table 2). The diagnostic confidence for lesion detection was assessed using a 4-point grading system as follows: 0, no diagnostic value; 1, suboptimal diagnostic utility; 2, moderate diagnostic reliability; 3, high diagnostic certainty.

**Table 2:** Scale for subjective assessment of image quality

Grading	Overall image quality	Truncation artifact	Motion artifact	Cerebrospinal fluid flow artifact	Vascular pulsation artifact	Significance of lesions
1	Unacceptable	Unable to meet diagnostic requirements				Invisible
2	Poor	Severe artifact (significantly impairing diagnose)				Blurred but visible
3	Acceptable	Moderate artifact (mild interfering diagnose)				Acceptable
4	Good	Minor artifacts (not influencing diagnose)				Good
5	Perfect	No visible artifact				Perfect

### Objective image analysis

Quantitative image analysis was conducted by an observer with seven years of MRI experience at a post-processing workstation (uWS MR, R005) and subsequently reviewed by another observer with twelve years of MRI experience. The signal intensities (SI) of the bilateral gray matter (GM), white matter (WM), and cerebrospinal fluid (CSF) were calculated using manually depicted regions of interest (ROI) of the appropriate size on the T2WI-PI images. The ROIs for GM and white matter WM were manually delineated on the frontal lobes. The ROIs for CSF were manually delineated on the lateral ventricles, avoiding the choroid plexus. The ROIs for T2WI-ACS and T2WI-ACS-DLR images are obtained synchronously with position and size unchanged. The image noise was defined as the standard deviation ($\:{{\upsigma\:}}_{noise}$) calculated from the ROI measurements in the four corners of the image’s background area. Finally, the formulas used to calculate the SNR and CNR were as follows:

$$SNR=\frac{{{\mu _{tissue}}}}{{{\sigma _{noise}}}}$$

$$CNR={{\left| {({\mu _{tissue1}} - {\mu _{tissue2}})} \right|} \mathord{\left/ {\vphantom {{\left| {({\mu _{tissue1}} - {\mu _{tissue2}})} \right|} {\sqrt {\sigma _{{_{{tissue1}}}}^{2}+\sigma _{{_{{tissue2}}}}^{2}} }}} \right. \kern-0pt} {\sqrt {\sigma _{{_{{tissue1}}}}^{2}+\sigma _{{_{{tissue2}}}}^{2}} }}$$

where $\:{\mu\:}_{tissue}$ is the meaning of SI of WM, GM or CSF, and $\:{{\upsigma\:}}_{noise}$ is the variance of background.

### Statistical analysis

Statistical analyses were conducted using SPSS 26.0 (IBM Corp, Armonk, NY, USA). The Shapiro-Wilk test was employed to determine if the data followed a normal distribution, for continuous data with a normal distribution are reported as the means ± standard deviation (SD), whereas non-normally distributed data are expressed as the median and interquartile range (IQR). For non-normally distributed data, multiple comparisons were performed using related samples Friedman’s two-way analysis, followed by the Bonferroni correction. For normally distributed data, multiple comparisons were performed using the repeated measures ANOVA, followed by the Least Significant Difference post hoc test. Interreader agreement for subjective image evaluation was measured with Cohen’s Kappa coefficient. The level of agreement was interpreted as follows: poor, ≤ 0.20; fair, 0.21–0.40; moderate, 0.41–0.60; good, 0.61–0.80; and excellent, 0.81-1.00. A *P*-value < 0.05 was considered significant.

## Results

### Patient characteristics and acquisition time

In this study, 104 patients with suspected brain disease received an MRI examination. Among them, six patients were excluded due to their sequences not being synchronized. Finally, a total of 98 patients (59 female and 39 male; mean age, 54.7 years ± 14.8[SD]) were included and analyzed. In terms of T2WI acquisition time, as shown in Table 1, the acquisition time for the T2WI-PI was 159 s, while the acquisition time for both T2WI-ACS and T2WI-ACS-DLR was 35 s.

### Subjective image evaluation

The results of the two observers on the overall image quality, truncation artifacts, motion artifacts, cerebrospinal fluid pulsation artifacts, vascular pulsation artifacts, and the significance of lesions are shown in Table 3. A good agreement (κ = 0.74) was observed for T2WI-ACS overall image quality assessments, whereas excellent agreement (κ > 0.90) was achieved across all other subjective evaluations. There were significant differences in the overall image quality among the T2WI-ACS (4, [4, 4]), T2WI-ACS-DLR (5, [5, 5]), and T2WI-PI (5, [5, 5]) sequences (*P* < 0.001). Pairwise comparisons revealed that the T2WI-ACS-DLR sequence demonstrated superior overall image quality compared to the T2WI-ACS sequence (*P* < 0.001), while no significant difference in overall image quality was observed between the T2WI-ACS-DLR and T2WI-PI sequences (*P* > 0.05). Images acquired with T2WI-ACS and T2WI-ACS-DLR showed almost the equivalent ratings to those of T2WI-PI images in terms of artifacts and the significance of lesions (*P* > 0.05). Images acquired with T2WI-ACS (3, [3, 3]) showed almost the equivalent ratings to those of T2WI-ACS-DLR (3, [3, 3]), and T2WI-PI (3, [3, 3]) images in terms of diagnostic confidence in lesion detection for two readers (*P* > 0.05).

**Table 3:** Subjective image analysis for ACS, ACS-DLR, and PI

Subjective evaluation	Reader	ACS	ACS-DLR	PI	*P*
Overall image quality	1	4(4,4)	5(5,5)	5(5,5)	< 0.001
	2	4(4,4)	5(5,5)	5(5,5)	< 0.001
Interreader agreement		0.74	0.95	0.91	
Truncation artifact	1	5(5,5)	5(5,5)	5(5,5)	0.368
	2	5(5,5)	5(5,5)	5(5,5)	0.697
Interreader agreement		0.99	0.98	0.98	
Motion artifact	1	5(5,5)	5(5,5)	5(5,5)	0.957
	2	5(5,5)	5(5,5)	5(5,5)	0.738
Interreader agreement		0.93	0.94	0.91	
Cerebrospinal fluid flow artifact	1	5(5,5)	5(5,5)	5(5,5)	0.135
	2	5(5,5)	5(5,5)	5(5,5)	0.097
Interreader agreement		0.98	1	0.99	
Vascular pulsation artifact	1	5(5,5)	5(5,5)	5(5,5)	0.050
	2	5(5,5)	5(5,5)	5(5,5)	0.135
Interreader agreement		0.97	0.99	1	
Significance of lesions	1	5(5,5)	5(5,5)	5(5,5)	0.497
	2	5(5,5)	5(5,5)	5(5,5)	0.368
Interreader agreement		0.98	0.99	0.98	
Diagnostic confidence	1	3(3,3)	3(3,3)	3(3,3)	0.097
	2	3(3,3)	3(3,3)	3(3,3)	0.097
Interreader agreement		1	1	0.98	

Note. Data are expressed as median and interquartile range (IQR). ACS, artificial intelligence-assisted compressed sensing; DLR, deep learning-based reconstruction; PI, parallel imaging

### Objective image analysis

Quantitative data for SNR and CNR are summarized as the mean and standard deviation (SD) or median and interquartile range (IQR) in Table 4. The WM-SNR, GM-SNR, and CSF-SNR of T2WI-ACS-DLR (216.57 ± 30.10, 367.00 [337.13, 396.30], and 1047.81 ± 125.82, respectively) were significantly superior to those of both T2WI-ACS (129.58 ± 20.87, 219.38 [199.25, 243.28], and 623.77 ± 82.90, respectively) and T2WI-PI sequences (146.69 ± 21.13, 249.25 [217.90, 270.30], and 685.13 ± 102.18, respectively), respectively ( all *P* < 0.001). The WM-GM CNR, GM-CSF CNR, and WM-CSF CNR of T2WI-ACS-DLR sequences (7.18 [5.61, 8.80], 23.28 ± 6.87, and 34.26 [27.77, 41.12], respectively) were significantly higher than those of T2WI-ACS sequences (4.35 [3.66, 4.84], 15.51 ± 2.95, and 21.88 [19.10, 23.47], respectively), respectively (*P* < 0.001), while there was no significant difference compared to the T2WI-PI sequences (7.35 [5.79, 9.07], 24.86 ± 7.47, and 36.56 [28.92, 44.66], respectively) (all *P* > 0.05).

**Table 4:** Pairwise comparisons of objective assessments among the three sequences

SNR		*p*	CNR		*p*
WM(ACS)	129.58 ± 20.87	<0.001^a^	WM-GM(ACS)	4.35(3.66,4.84)	<0.001^a^
WM(DLR)	216.57 ± 30.10	<0.001^b^	WM-GM(DLR)	7.18(5.61,8.80)	<0.001^b^
WM(PI)	146.69 ± 21.13	<0.001^c^	WM-GM(PI)	7.35(5.79,9.07)	0.432^c^
GM(ACS)	219.38(199.25,243.28)	<0.001^a^	GM-CSF(ACS)	15.51 ± 2.95	<0.001^a^
GM(DLR)	367.00(337.13,396.30)	<0.001^b^	GM-CSF(DLR)	23.28 ± 6.87	<0.001^b^
GM(PI)	249.25(217.90,270.30)	<0.001^c^	GM-CSF(PI)	24.86 ± 7.47	0.353^c^
CSF(ACS)	623.77 ± 82.90	<0.001^a^	WM-CSF(ACS)	21.88(19.10,23.47)	<0.001^a^
CSF(DLR)	1047.81 ± 125.82	<0.001^b^	WM-CSF(DLR)	34.26(27.77,41.12)	<0.001^b^
CSF(PI)	685.13 ± 102.18	<0.001^c^	WM-CSF(PI)	36.56(28.92,44.66)	0.852^c^

Note. Continuous values are expressed as mean ± SD or median and interquartile range (IQR). WM, white matter; GM, grey matter; CSF, cerebrospinal fluid. ACS, artificial intelligence-assisted compressed sensing; DLR, deep learning-based reconstruction; PI, parallel imaging. a, comparison between T2WI-ACS and T2WI-ACS-DLR; b, comparison between T2WI-ACS and T2WI-PI; c, comparison between T2WI-ACS-DLR and T2WI-PI

## Discussion

This present study integrated a novel DLR technique into an ACS-accelerated T2WI sequence using 5.0T MRI to enable rapid acquisition of brain T2WI sequences. The performance of this innovative approach was systematically compared with conventional PI-accelerated acquisition sequences through comprehensive evaluations, including subjective image quality assessment, objective quantitative metrics analysis, and acquisition time measurement. The results revealed that both T2WI-ACS and T2WI-ACS-DLR sequences achieve a remarkable 78% reduction in acquisition time compared to traditional T2WI-PI sequences (35 s vs. 159 s, respectively). Notably, the T2WI-ACS-DLR sequence not only preserved this significant time efficiency but also provided superior overall image quality, characterized by the highest SNR while maintaining comparable CNR to conventional T2WI-PI sequences.

**Fig. 1:**
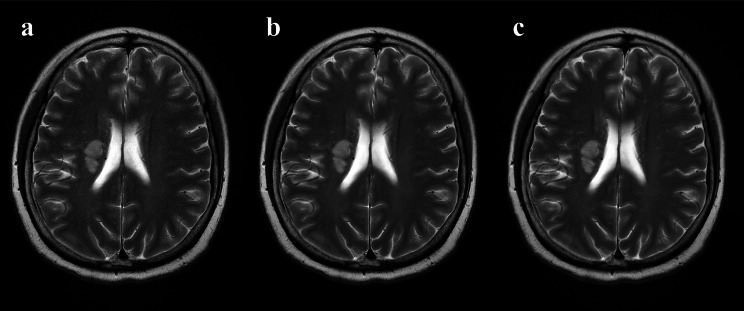
A case example from a 60-year-old woman with acute ischemic stroke. a, T2WI-ACS; b, T2WI-ACS-DLR; c, T2WI-PI. The noise in T2WI-ACS (**a**) is quite noticeable. After applying DLR technology (**b**), image quality improvement is comparable to that of PI (**c**)

Monch, S, et al. [17] assessed the effects of CS on the image quality and acquisition speed of conventional brain MRI, showing a median scan time reduction of 29.3% (range: 0.0-58.4%). This finding is further supported by a comparative study in head and neck MRI, where the combined application of CS and PI resulted in significantly shorter scan time (83.5 ± 11.0 s) compared to PI alone (173.0 ± 54.4 s) [5]. In contrast to previous studies that primarily focused on scan time reduction using traditional methods, this study reduced the number of excitations (NEX) from 1.5 (T2WI-PI) to 1 and employed an ACS technique (T2WI-ACS) that integrates artificial intelligence with three acceleration techniques (CS, PI, and HF). This approach achieved a 78% acquisition time reduction, consistent with recent advancements in rapid imaging techniques [11]. Theoretically, keeping other parameters constant, reducing the NEX by one-third proportionally decreases the acquisition time by one-third (33.3%), while the ACS technology further reduces it by 44.6%. This fact is not surprising when considering the fundamental characteristics of NEX and ACS. NEX is a factor that affects the acquisition time, as NEX decreases, the acquisition time correspondingly decreases, but also does the SNR [18]. By utilizing deep learning on prior knowledge, recent advancements in AI algorithms enable faster imaging techniques to overcome the limitations of CS-related noise and artifacts, PI-associated SNR reduction, and HF-induced artifacts from incomplete k-space data [19]. Despite these technological advancements, our visual image evaluation revealed an interesting observation. While the T2WI-ACS sequence received consistent median scores of 4 for overall image quality, nearly all other parameters were rated as 5 by observers. This discrepancy, as illustrated in Fig. 1, correlates with the quantitative finding that T2WI-ACS exhibited lower SNR compared to both T2WI-PI and T2WI-ACS-DLR sequences. These findings may be attributed to the combined effects of NEX reduction and ACS acceleration, suggesting a potential trade-off between acquisition speed and certain image quality parameters that warrants further investigation.

While our study demonstrates the successful implementation of the ACS method for achieving ultra-fast brain scans, it is noteworthy that the T2WI-ACS sequence exhibited inferior SNR and CNR compared to conventional PI sequences, as illustrated in Table 4. These quantitative metrics, widely recognized as crucial indicators for assessing MR image quality, highlight the inherent challenge in balancing acquisition speed with diagnostic image quality in clinical practice [20]. However, the rapid advancement of AI in medical imaging, particularly through DLR algorithms, has provided promising solutions to this dilemma [21–23]. Our protocol incorporates a deep CNN model for DLR intelligent reconstruction, which effectively addresses the limitations of traditional acceleration methods by implementing intelligent noise reduction and fidelity enhancement. This innovative integration of DLR technology within the ACS framework has yielded significant improvements in both SNR and CNR metrics compared to conventional PI techniques. The clinical relevance of these advancements is particularly evident in the enhanced visualization of anatomical structures and pathological features, facilitating more accurate diagnostic interpretation. The practical advantages of our approach extend beyond image quality enhancement. The T2WI-ACS-DLR sequence demonstrates remarkable efficiency in reconstruction time, enabling immediate availability of reconstructed images upon scan completion. This dual benefit of reduced acquisition time and improved image quality successfully tackles two critical aspects of clinical MRI, encompassing both patient comfort and diagnostic efficacy. The shortened scan duration not only minimizes patient discomfort but also reduces the likelihood of motion artifacts while potentially increasing patient throughput [24].

**Fig. 2:**
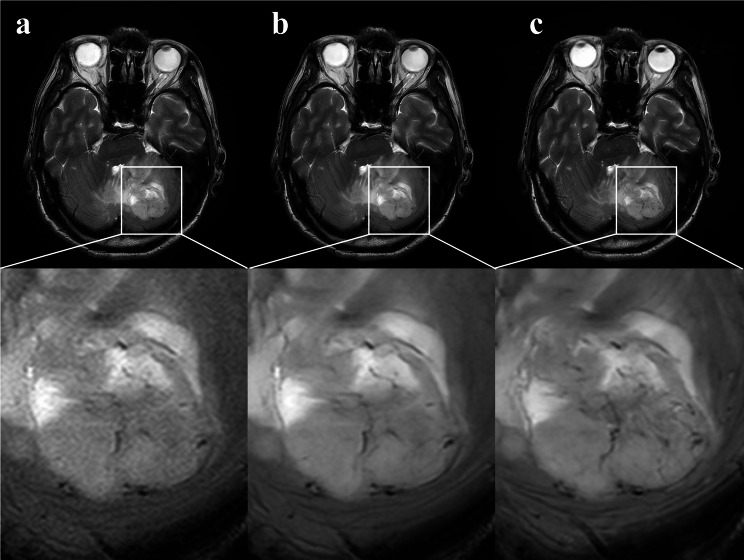
A case example from a 64-year-old man with glioblastoma. **a**, T2WI-ACS; **b**, T2WI-ACS-DLR; **c**, T2WI-PI. T2WI-ACS-DLR (b) and T2WI-PI (c) provide a clearer representation of the internal details of the tumor, as well as the distinct boundaries and contrasts of the lesions compared to T2WI-ACS (a)

**Fig. 3:**
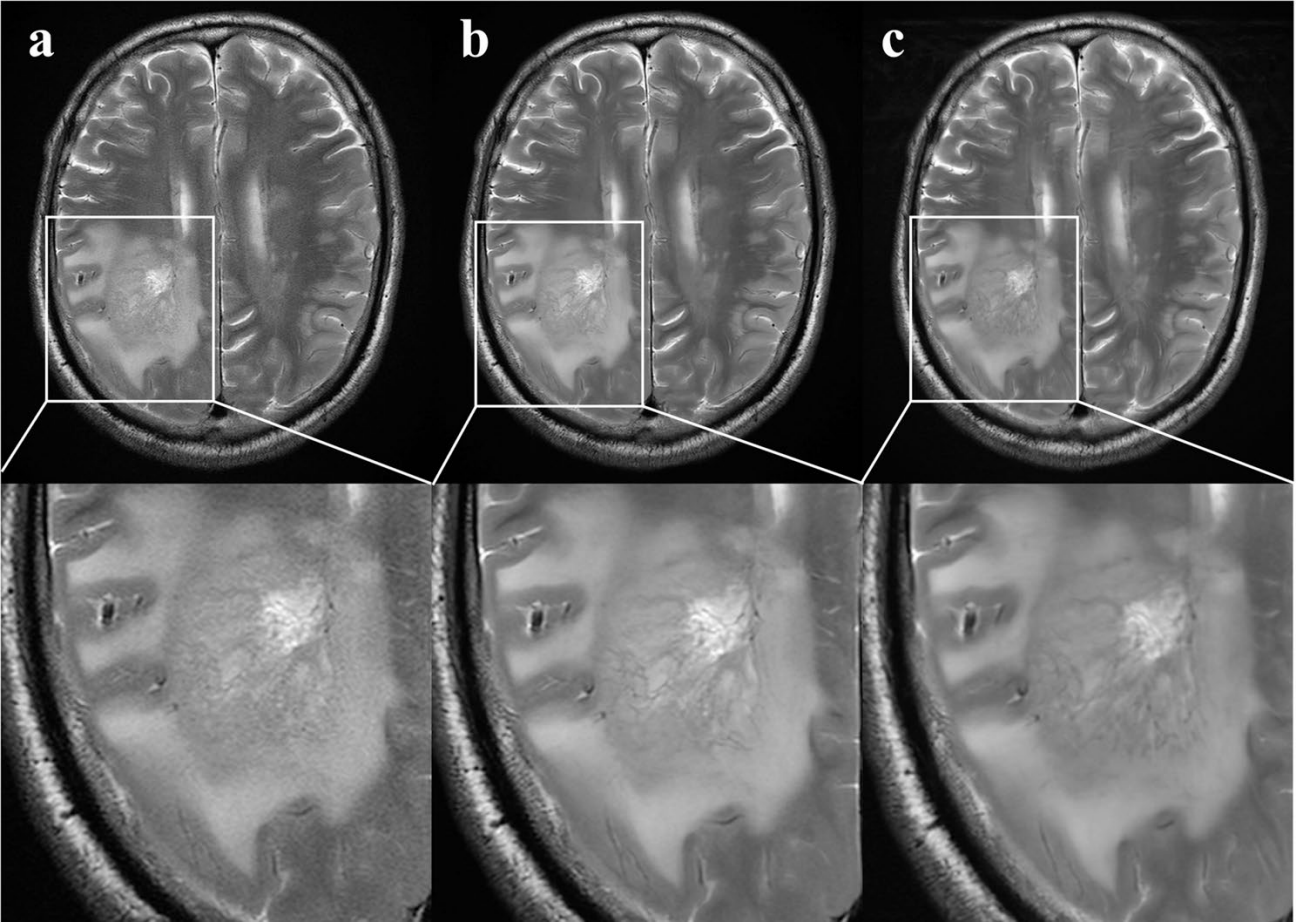
A case example from a 77-year-old man with glioblastoma. **a**, T2WI-ACS; **b**, T2WI-ACS-DLR; **c**, T2WI-PI. T2WI-ACS (a) has a tumor visibility score of 4, while T2WI-ACS-DLR (b) and T2WI-PI (c) both have tumor visibility scores of 5 with richer internal details

For neuroimaging applications, dipole array configurations facilitate comprehensive brain coverage with extended longitudinal field-of-view, ensuring the inclusion of cerebellar structures. Typically, the number of transmit channels ranges from 8 to 16, depending on the radio frequency hardware of the scanner [25]. Image quality and acquisition speed are fundamentally governed by the coil’s channel density, internal element count, and spatial distribution of these elements. In a previous study [26], the 38-channel array achieved the highest SNR in the center of the brain, being 1.27 times greater than the 16-channel array, and up to 2.3 times higher than the 8-channel array. The 48-channel head coil employed in this study inherently benefits from high spatial encoding capacity, contributing to superior SNR and parallel imaging at 5.0T. This enables shorter acquisition time or higher resolution acquisitions compared with 3.0T. Moreover, 5.0T provides a higher intrinsic SNR than 3.0T, enabling higher spatial resolution and improved visualization of small anatomical structures (e.g., microvasculature) [27]. Compared to 7.0T, 5.0T mitigates challenges such as B1 field inhomogeneity, SAR limitations, and susceptibility artifacts, which limit its broader clinical application. While 5.0T systems are not yet widespread, their design bridges the gap between research-focused 7.0T and clinical 3.0T scanners, facilitating robust imaging protocols while maintaining patient safety and comfort. The clinical superiority of our approach is further substantiated by the comparative analysis of lesion visualization across the three sequences, as presented in Figs. 2 and 3. The T2WI-ACS-DLR sequence consistently demonstrates an enhanced depiction of tumor internal architecture, improved lesion boundary definition, and superior tissue contrast. These findings align with recent advancements in accelerated MRI techniques [15, 16, 28], strongly supporting the clinical applicability and diagnostic potential of our proposed method.

Despite the outstanding performance of the T2WI-ACS-DLR in patients with suspected intracranial lesions, there are several limitations in this study. First, due to the single-center and small sample design, it is necessary to conduct multi-center research in future studies to further validate the research results after expanding the data. Second, this study did not perform a detailed analysis of a specific type of intracranial lesions, as the heterogeneity among different types of lesions may potentially influence the measurement outcomes. Future investigations should focus on certain specific diseases to explore more refined application scenarios. Notably, the study of ACS combined with DLR acceleration technology in patients with stroke will be interesting, as it may provide more time to salvage the ischemic penumbra in cases of acute ischemic stroke. Third, this study only utilized ACS combined with DLR to acquire images at a single magnetic field strength of 5.0T. Future research could investigate the impact of different magnetic field strengths on image quality, acquisition time, and the performance of ACS and DLR.

In conclusion, the combination of ACS and DLR technology enables rapid scanning of brain T2WI while achieving higher SNR and comparable CNR compared to the PI sequence, confirming the feasibility in clinical applications at 5.0T.

## Data Availability

No datasets were generated or analysed during the current study.
